# Gamma ray shielding capability of flexible silicone rubber composites reinforced with recycled CRT glasses

**DOI:** 10.1038/s41598-025-10920-3

**Published:** 2025-07-21

**Authors:** Doaa A. Elsayed, Mohamed Elsafi, Esraa H. Abdel-Gawad, Shoaa M. Al-Balawi, M. I Sayyed, Ibrahim H. Saleh

**Affiliations:** 1 Environmental Studies Department, Institute of Graduate Studies and Research, Alexandria University, Alexandria, 21511 Egypt; 2 Physics Department, Faculty of Science, Alexandria University, Alexandria, 21511 Egypt; 3 General Science Program-Deanship of Support Studies, Alasala University, Dammam, Saudi Arabia; 4 Department of Physics, Faculty of Science, Isra University, Amman, Jordan

**Keywords:** CRT-glass, Mechanical, Radiation absorption ratio, Shielding, Silicon rubber, Thermal, Physics, Nuclear physics, Experimental nuclear physics

## Abstract

With a focus on extending the horizons of combining electronic waste (e-waste) with different materials for creating efficient, affordable, eco-friendly, and sustainable radiation shielding composites, the present study investigates the radiation shielding performance of different composites made of silicon rubber and e-waste glass. Six samples were prepared by mixing different amounts of powdered cathode ray tube (CRT) glass (0–50%) with silicon rubber (100–50%). The linear attenuation coefficients (LAC’s) of the prepared samples were experimentally measured using a high-purity germanium detector and varying energy gamma sources. Other radiation shielding parameters such as mass attenuation coefficient (MAC), transmission factor (TF), mean free path (MFP), half value layer (HVL) and radiation attenuation ratio (RAR) were calculated. The sample with the highest CRT content (SR-CRT-50) showed the highest efficiency superior to the rest of the prepared samples with maximum LAC (2.051 cm^−1^), maximum MAC (1.1890 cm^2^/g), minimum HVL (0.338 cm), and minimum TF (0.357) at the lowest energy level of 0.060 MeV. The additive of waste CRT glass to the matrix also improve the mechanical and thermal properties of composites, and accordingly the weight loss at about 620 ± 15 °C was 70.09%, 53.17%, and 32.37% for SR-CRT-0, SR-CRT-30, and SR-CRT-50, respectively. Clearly, this study demonstrated that adding waste CRT glass to the silicone rubber polymer increased its performance as a shield against ionizing photons.

## Introduction

Radiation shielding is a crucial practice in various sectors such as medical imaging and treatment, nuclear industry, and aerospace to safeguard individuals and equipment from harmful radiation exposure. Efficient shielding should lead to significant energy loss of ionizing radiation within a certain thickness. The selection of the shielding material depends on the type of radiation, energy levels, cost considerations, and the specific application. Radiation shielding materials are categorized into glasses^1,2^, alloys^3^, ceramics^4,5^, concretes^6^, and polymers^7,8^. Mixing two or more different materials, or in other words, using composite shields, can augment the effectiveness of radiation shielding in specific situations^9^.

Using electronic waste (e-waste) as a basic component in the composition of different radiation shielding composites can lead to financial and environmental advantages. Recycling e-waste for being used in preparing shielding composites can decrease the demand for new raw materials i.e. conserve resources. This approach is considered cost-effective as it lowers production costs. Additionally, recycling e-waste for shields manufacturing can also reduce disposal, decreases landfilling and mitigates environmental pollution^10–12^. The modern technology in display devices has avoided the closed-loop recycling of a special part of e-waste called the cathode ray tube (CRT) glass to avoid lead leaching possibility. Hence, CRT glass accumulates in the environment as unwanted materials. However, the reason which makes CRT improper for disposal and closed-loop recycling makes it a good choice for effective radiation attenuation^13–15^. CRT glass consists of several oxides of metals including lead, barium, and strontium, etc. Lead and its oxides were proven to be highly effective materials for radiation shielding owing to its high density and ability to absorb and scatter ionizing radiation effectively^16^. For barium, it is known for its ability to attenuate photons. The presence of barium oxide (BaO) in a glass system leads to enhancing the shielding performance of a glass network against radiation according to the studies of Aloraini and his research team^17^, also, Aboalatta and his colleagues^18^. For strontium oxide (SrO), it was found to boost the mass attenuation coefficient in the study of Mhareb and his fellow researchers^19^. In more recent studies, Al-Buriahi, Al-zahrani and Basha with their research teams proved great shielding performance of recycled CRT with Bi_2_O_3_ addition strategies^20–23^. Additionally, Alrowaili et al*.*, showed that recycled waste CRT glasses doped with Li_2_O and Y_2_O_3_ has enhanced radiation shielding performance^24^. Moreover, Kurtulus et al*.*, studied radiation shielding parameters for CRT-derived glass systems containing CoO^25^.

Considering the idea of making composite shields (made of two different materials), previous studies have been focusing on integrating CRT from e-waste with concrete shields for a decade already^12,26–31^. The literature review has prompted us to extend the ongoing research by investigating other radiation shielding composites like CRT with polymers. Polymers are multipurpose materials that are easy to mold for the several applications. One of the most promising polymers is silicon rubber (SR). SR has showed its potential to be utilized for radiation shielding applications e.g. medical, industrial, and aerospace. SR is known for its great flexibility; this feature has a wide range of uses, especially in the medical sector where a snug fit is required such as in medical imaging equipment and protective gears^32^. In the research of Lestari et al*.*, SR has been evaluated as a breast shields for thoracic CT scans; it offered effective radiation protection while maintaining comfort for the patient^33^. Moreover, Zali et al., highlighted the use of SR doped with tungsten oxide in sealing windows, doors, and appliances in different radiation applications. Additionally, SR’s lightweight nature is remarkably beneficial in applications where weight is an essential factor, such as in aerospace and military settings^34,35^. Another significant benefit of SR is its compatibility with different fillers that can enhance its radiation shielding effectiveness. For instance, Alresheedi, Elsafi, Almutairi and Aloraini with their research teams have proved that the addition of heavy metal oxides like lead bismuth, tungesten, barium, and tin has been proven to enhance the gamma radiation attenuation performance of silicone rubber composites^36–39^.

This is where the idea of ​​the present research came from, combining silicon rubber with CRT glass (from recycled e-waste) that contains heavy metal oxides to form a novel flexible composite for different radiation shielding applications. To assess the radiation-interaction performance for the proposed composite, six samples of silicon rubber (as a matrix) combined with different concentrations of CRT glass (as a filler) were prepared and the radiation shielding parameters of the samples were measured beside the thermal analyses of the samples.

## Materials and methods

### Samples preparation

The composite samples were fabricated by mixing some waste cathode ray tube (CRT) glass with silicone rubber (SR) in different ratios. Liquid SR with its hardener was purchased from commercial lstore in Egypt, its density 1.192 g cm^3^. The waste glass for this research was acquired from a television monitor 14 inches.

**Fig. 1:**
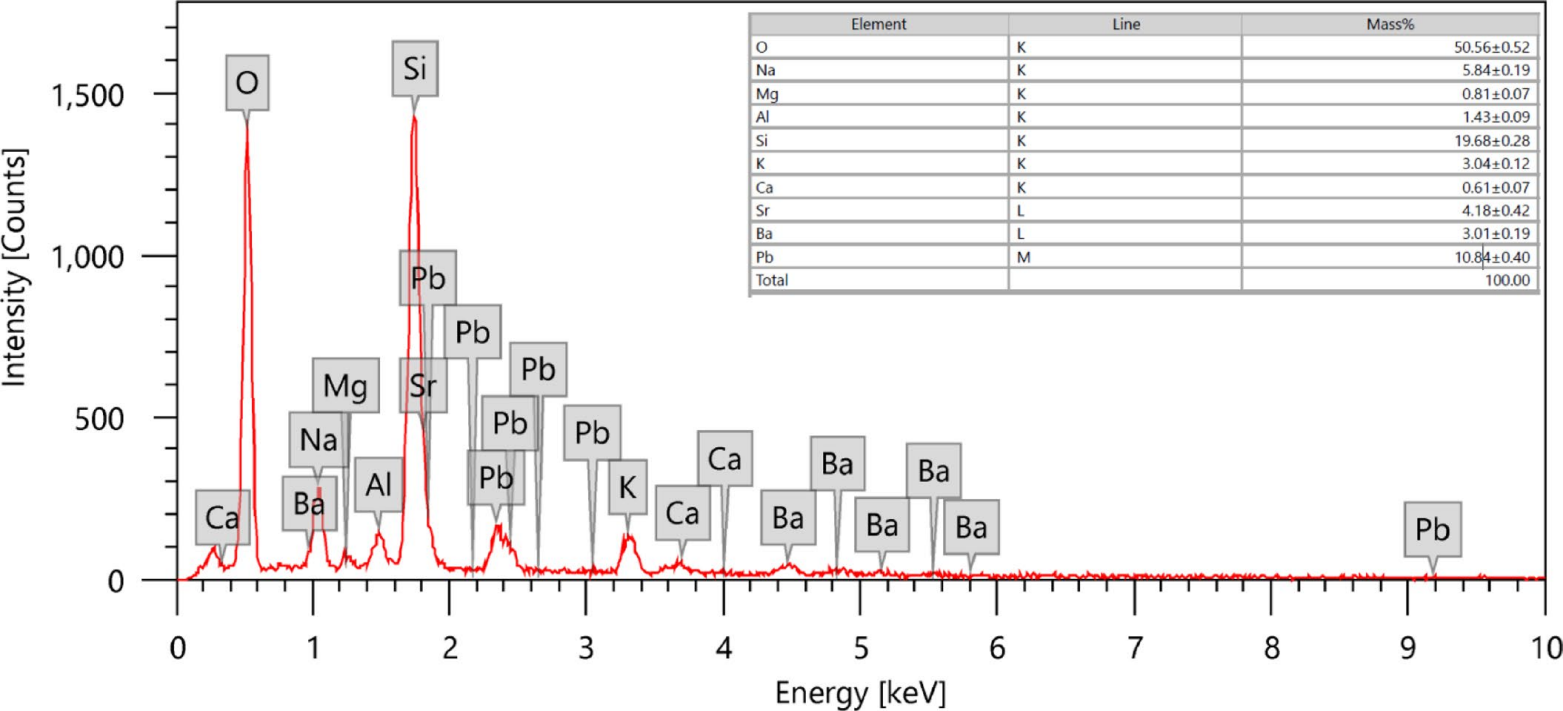
EDX analysis of cathode ray tube glass in the present work.

The fine powder of CRT glass was obtained by crushing it in a mixure. The chemical compositions of the waste glass sample were determined by the scanning electron microscope (SEM) equipped with Energy Dispersive X-ray spectroscopy (EDX) as shown in Fig. 1. From the chemical analysis of the CRT glass, the presence of silica in a large percentage and the presence of elements including lead, barium, and strontium was noted. Those elements are considered useful elements in radiation shielding applications due to their capabilities to attenuate ionizing radiation.

Six samples of SR-CRT were fabricated according to the mass percentages shown in Table 1. Liquid SR and CRT glass powder were added to a container and stirred well with the addition of a stabilizer until the produced composite became homogeneous then the composites were poured into molds and left to dry.

**Table 1:** The mixing ratios of fabricated SR composites and the determined densites.

Sample code	Silicone rubber (wt%)	CRT (wt%)	Density (g cm^−3^)
SR-CRT-0	100	0	1.192 ± 0.011
SR-CRT-10	90	10	1.323 ± 0.008
SR-CRT-20	80	20	1.405 ± 0.010
SR-CRT-30	70	30	1.497 ± 0.015
SR-CRT-40	60	40	1.603 ± 0.009
SR-CRT-50	50	50	1.725 ± 0.013

SEM or Scanning Electron Microscope (JSM-6010LV, JEOL) was used to characterize the distribution of shielding substances in composite samples. A beam accelerating voltage of 10 kV was applied to obtain the image of needed magnification.

Thermogravimetric analysis (TGA) measures the change in weight vs. temperature. For TGA measurement, tiny pieces (5–10 mg) were cut from the prepared composite samples and heated from 400 to 900 °C at a constant elevation of temperature 20 °C for 15 min in a nitrogen atmosphere. The equipment Model TGAQ50 was used to test the samples.

The mechanical properties of the prepared composite samples were assessed through stress–strain testing. The stress–strain curves were obtained using Generic Compression stress vs. strain device, which allowed the evaluation of mechanical performance by measuring the stress (MPs) at different strains and the overall elasticity of the materials.

**Fig. 2:**
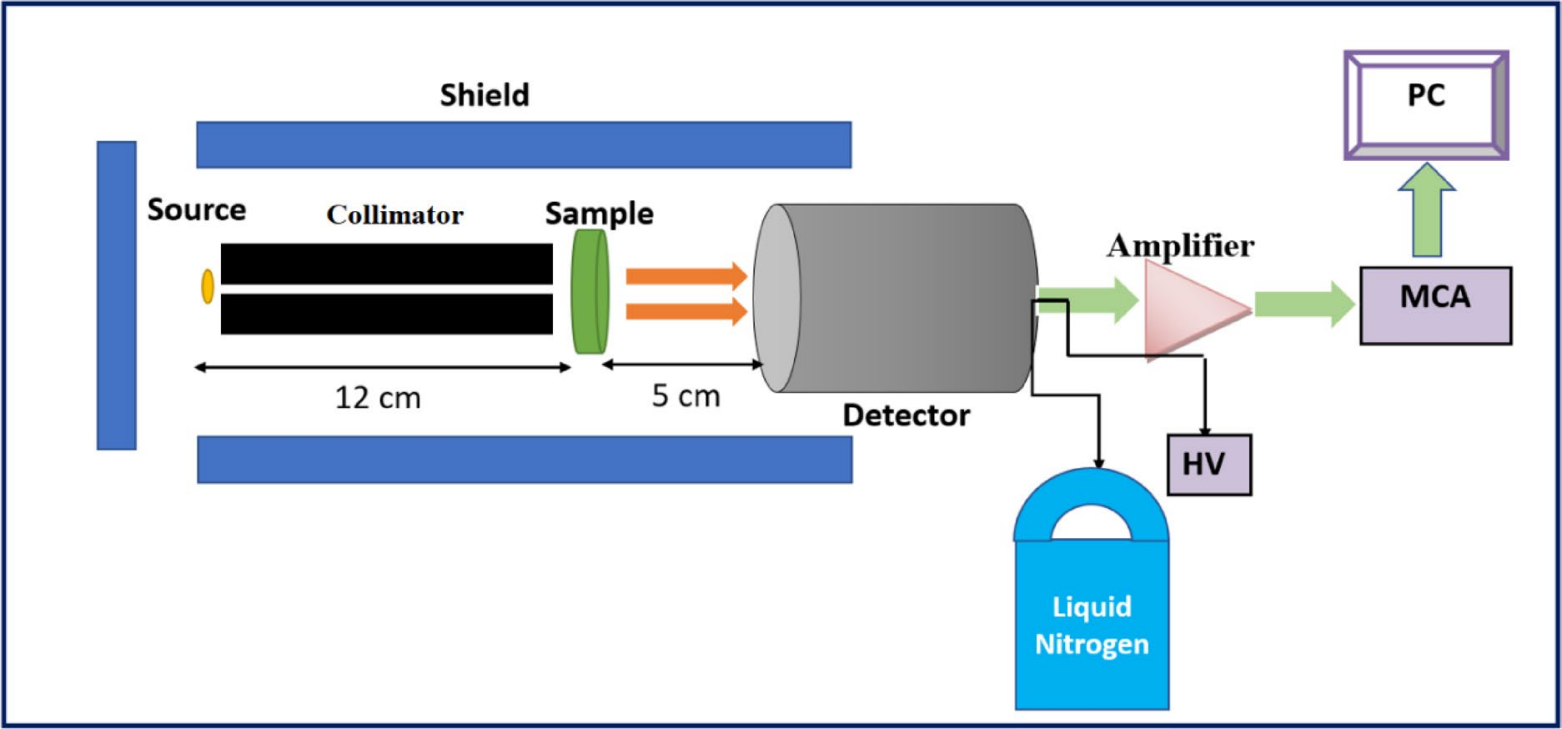
Geometry of experimental attenuation measurement.

**Fig. 3:**
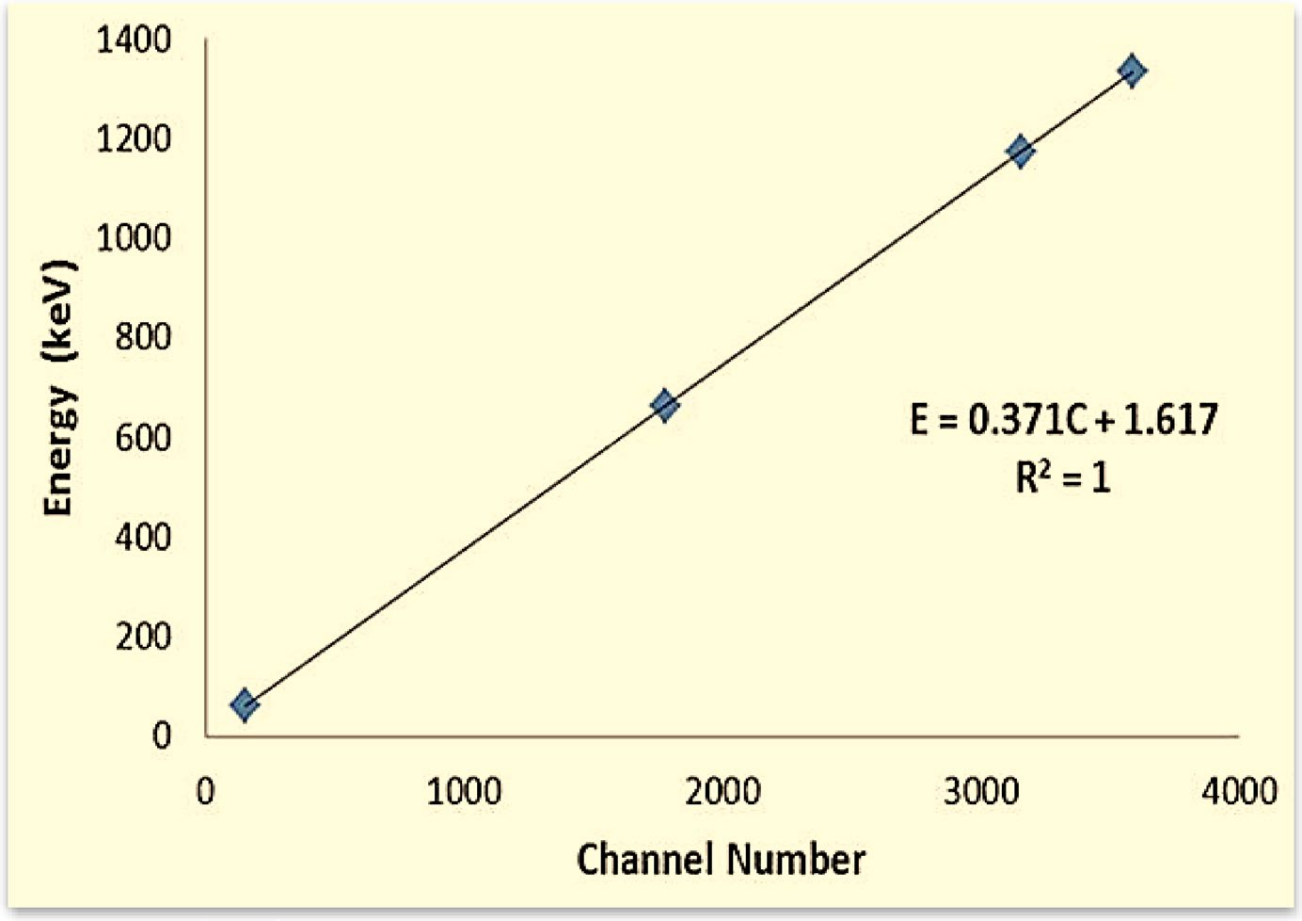
The energy calibration of the detector.

The radiation attenuation measurements of the fabricated composite samples (SR-CRT) were conducted experimentally by HPGe detector with relative efficiency 24% and energy resolustion 1.69 keV at 1.333 MeV and three diverse gamma sources to get wide range of energy (1.173 & 1.333 MeV from Co-60, 0.6617 MeV from Cs-137, and 0.0595 MeV from Am-241). The measuring mechanism is shown in Fig. 2. Before performing measurements, the detector was calibrated as shown in Fig. 3 using these three-point sources, and the sample position between the detector and the gamma source was adjusted using materials with known attenuation coefficients. During a specific time, the peaks associated with the incident energy photons radiated from the source were attained. The area below these peaks was estimated using Genie-2000 software. The calculated area per measuring time represents the intensity of the incoming photon. The intensity in the absence of a composite sample (SR-CRT) was signified by the initial intensity (${I}_{0}$), while the transmitted intensity ($I$) signified the measured intensity when an SR-CRT sample was placed between the detector and the radiation source.

From the obtained measurements, the linear attenuation coefficient (LAC) for an SR-CRT sample of a thickness ($x$) can be calculated by^40,41^:

1 $$LAC=\frac{1}{x} ln\frac{{I}_{0}}{I}$$

The other attenuator factors, such as mass attenuation coefficient (MAC), transmission factor (TF), half value length (HVL), tenth value length (TVL), Mean free path (MFP), and radiation attenuation ratio (RAR) can be expressed by the following equations:

2 $$MAC = LAC/Density$$

3 $$TF=\frac{I}{{I}_{0}}$$

4 $$HVL=\frac{Ln 2}{LAC}$$

5 $$TVL=\frac{Ln 10}{LAC}$$

6 $$MFP=\frac{1}{LAC}$$

7 $$RAR\%=\left[1-\frac{I}{{I}_{o}}\right]\times 100$$

## Results and discussions

**Fig. 4:**
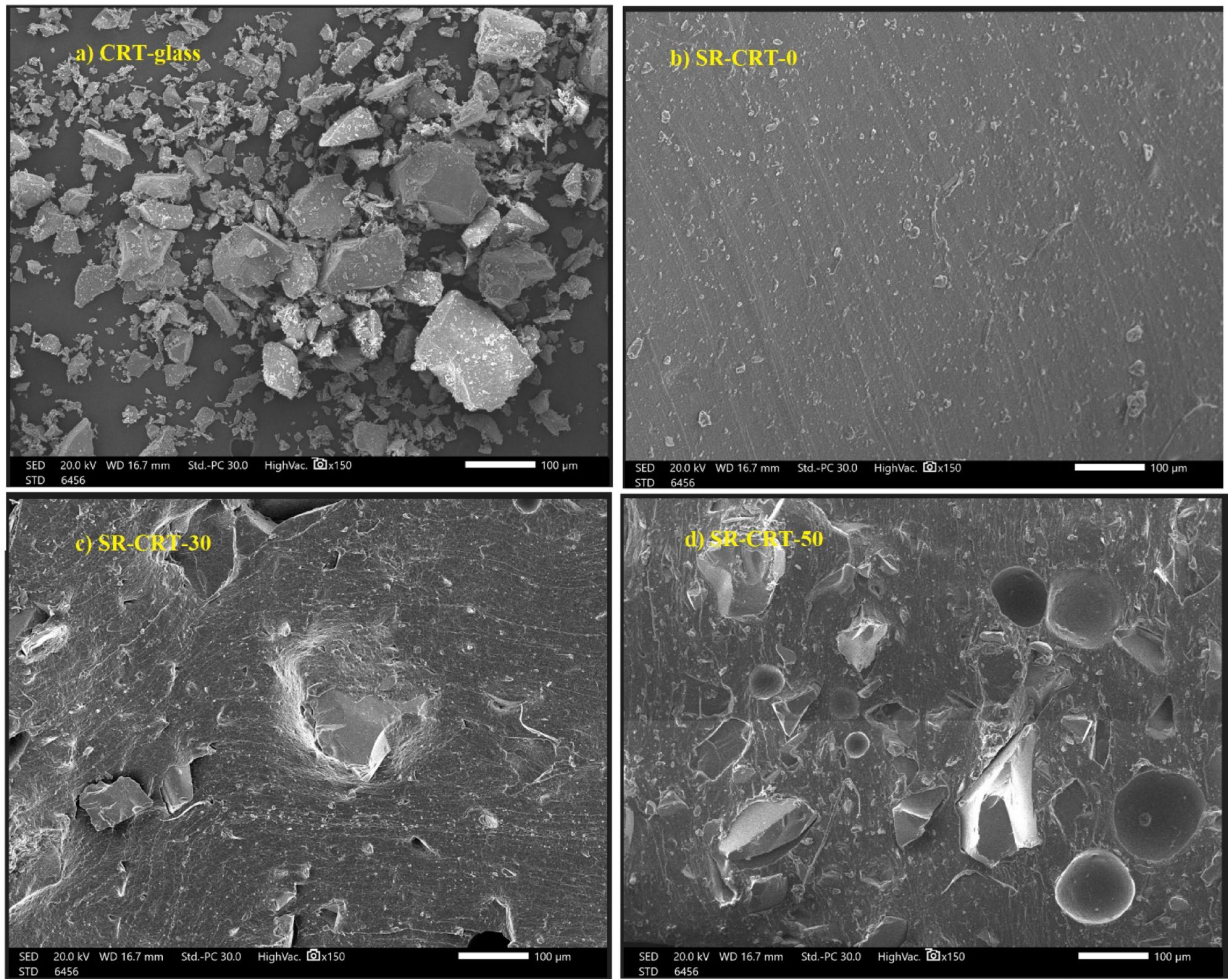
SEM images of (**a**) CRT-waste glass, (**b**) SR-CRT-0, (**c**) SR-CRT-30 and (**d**) SR-CRT-50.

Figure 4 shows the scanning electron microscope (SEM) images of the CRT-glass and pure silicone rubber (SR-CRT-0) samples separately, as well as the mixture in Fig. 4c,d. The figures show that there is a homogeneous distribution of particles within the rubber and that with the increase in the percentage of fillers within the rubber, the percentage of voids within the matrix decreases.

**Fig. 5:**
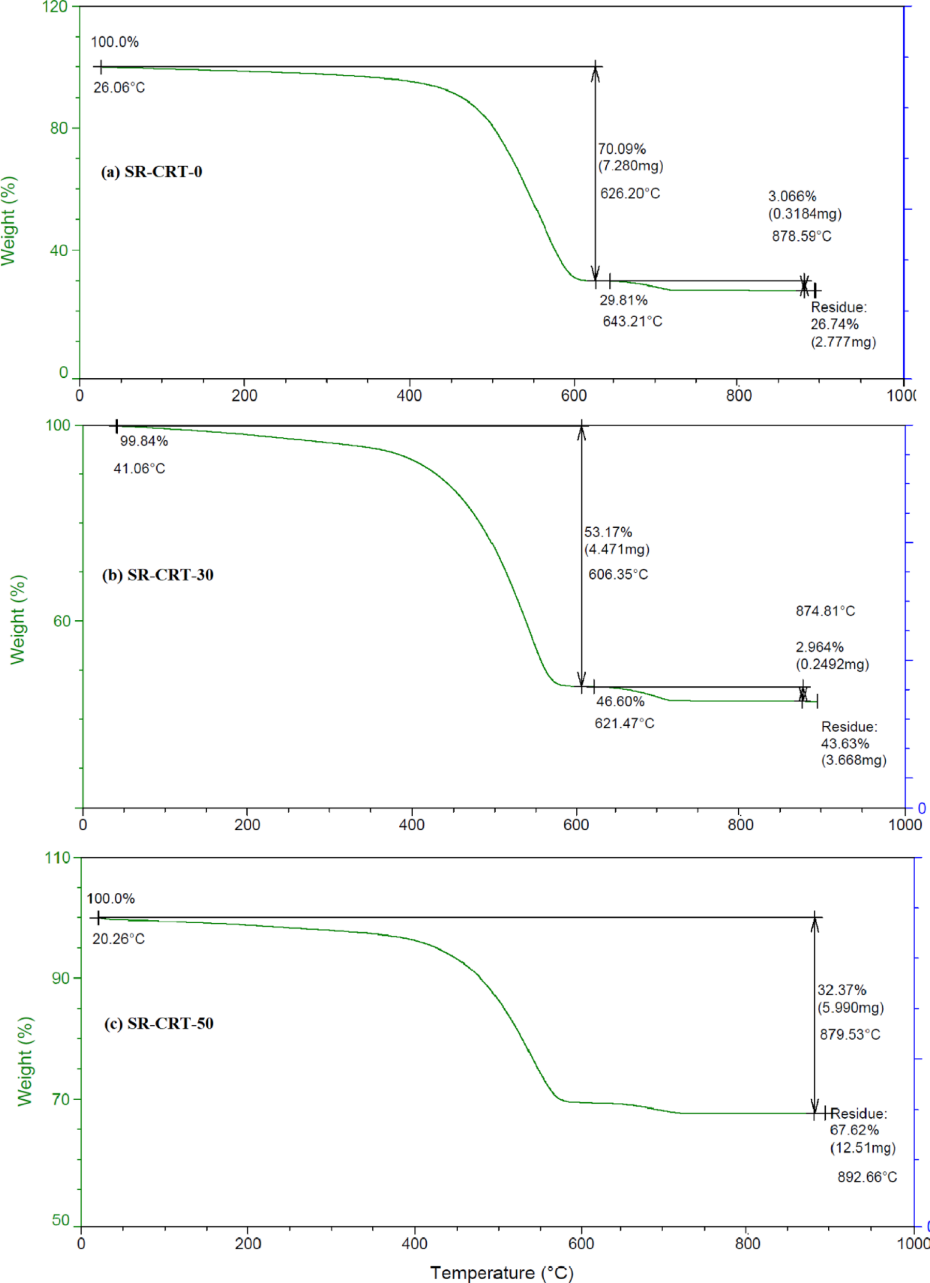
TGA of (**a**) CRT-waste glass, (**b**) SR-CRT-0, (**c**) SR-CRT-30 and (**d**) SR-CRT-50.

The TGA of the choosen composites (SR-CRT-0, SR-CRT-30 and SR-CRT-50) was analyzed as shown in Fig. 5. TGA thermogram of pure silicone rubber (SR-CRT-0) showed a 70.09% weight loss at the melting temperature 626.20 °C and 3.066% in 878.59 °C. No weight loss was observed prior to melting temperature at 453.52 °C, indicating its anhydrous nature. TGA thermogram (Fig. 4b) of composite sample SR-CRT-30 showed a 53.17% weight loss at the melting temperature 606.35 °C and 2.964% in 874.81 °C. No weight loss was observed prior to melting temperature at 480.76 °C, indicating its anhydrous nature. The melting peak is further followed by weight loss starting at a temperature of about 545.29 °C. TGA thermogram (Fig. 5c) of composite sample SR-CRT-50 showed a 32.37% weight loss at the melting temperature 636.70 °C. No weight loss was observed prior to melting temperature at 426.93 °C, indicating its anhydrous nature. The melting peak is further followed by weight loss starting at a temperature of about 541.13 °C. The results showed that adding CRT-glass waste improves the thermal properties of the silicone rubber, and the higher the percentage, the better the thermal properties.

**Fig. 6:**
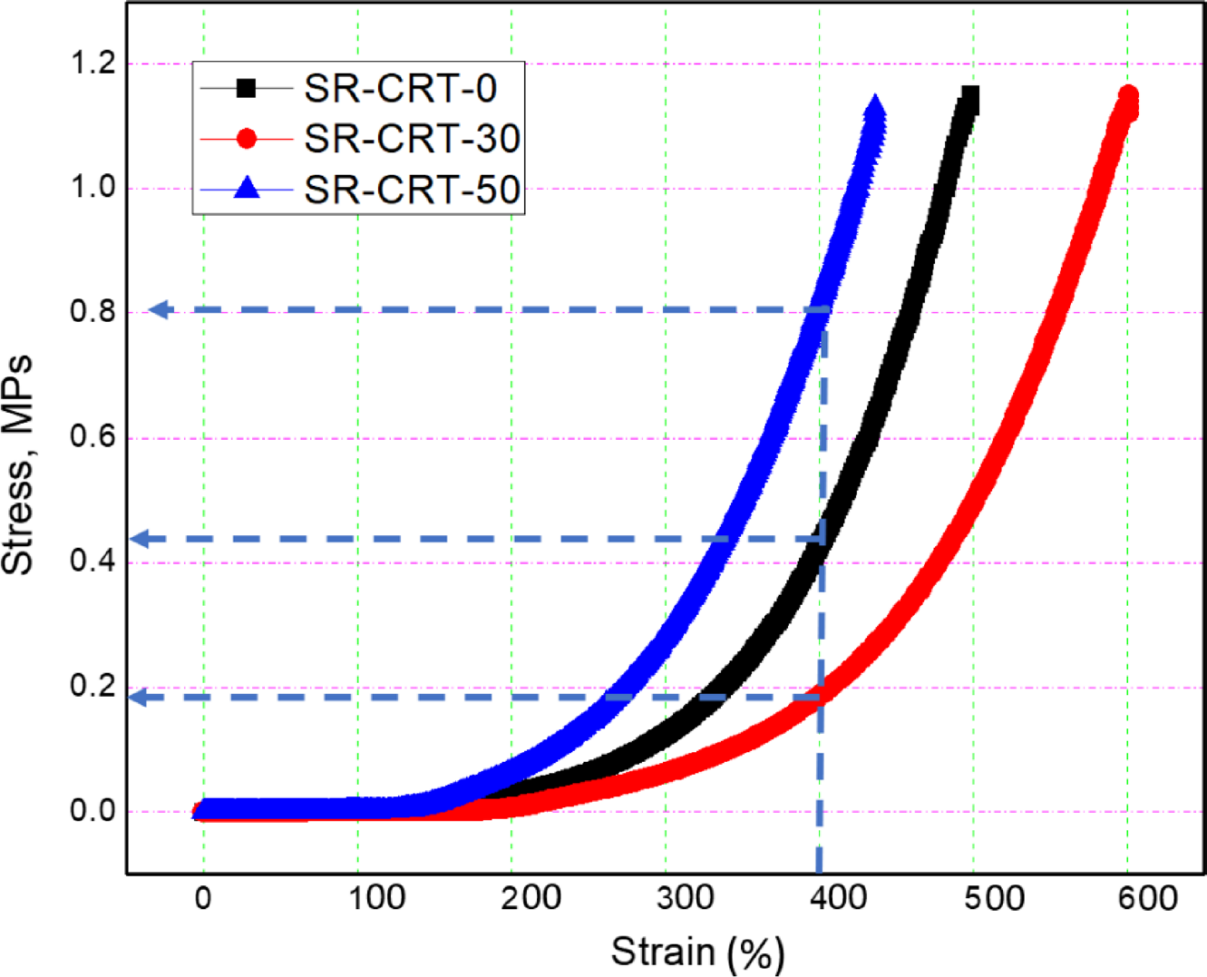
Stress–strain curve of SR-CRT composites.

The Stress–Strain relationship of SR-CRT-0, SR-CRT-30 and SR-CRT-50 has been determined as shown in Fig. 6. It was found that adding some CRT waste does not reduce its mechanical properties; 400% strain requires 0.44, 0.18 and 0.8 MPs for SR-CRT-0, SR-CRT-30 and SR-CRT-50, respectively.

**Fig. 7:**
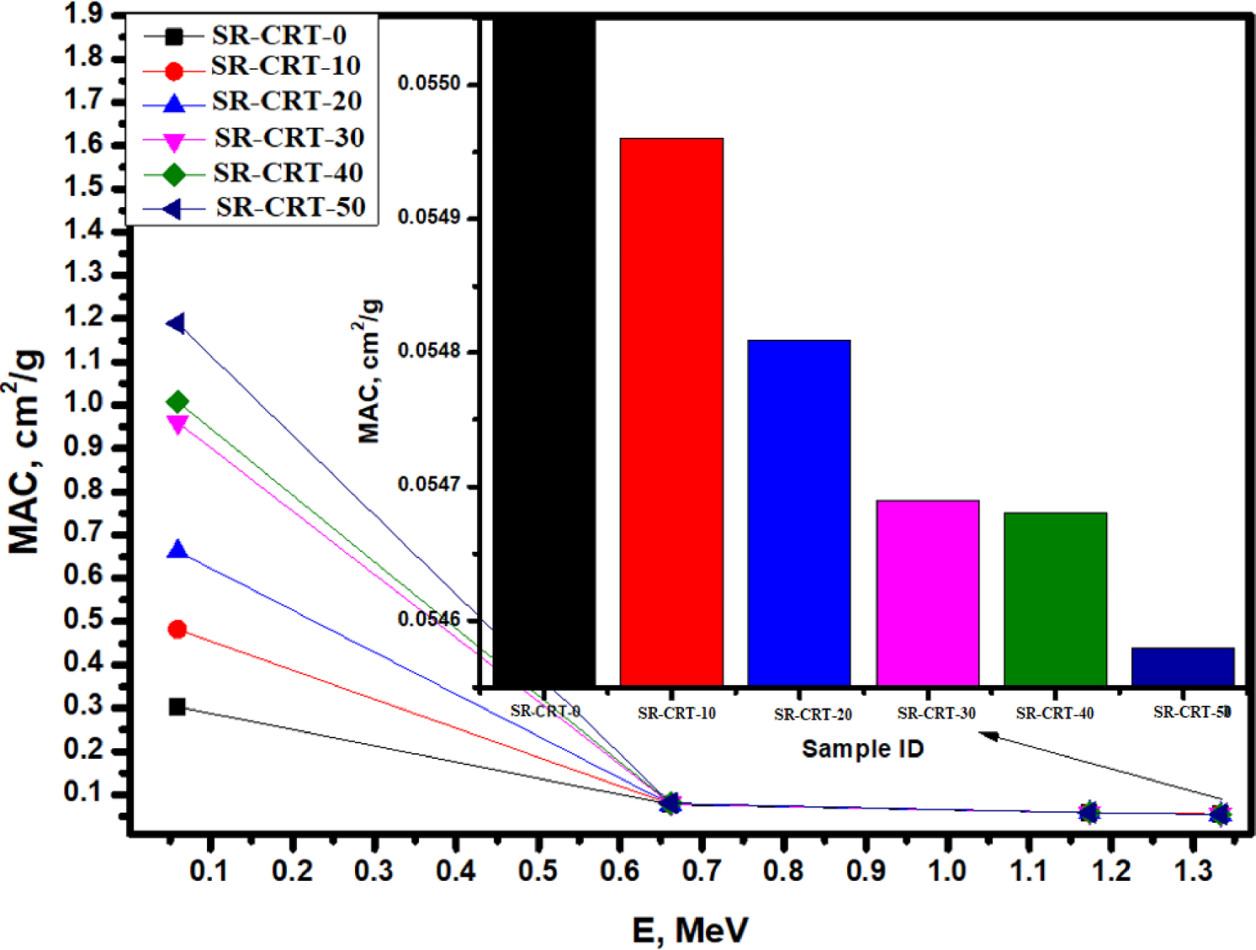
A chart of Mass Attenuation Coefficient MAC for all the studied samples as a function of Energy E in MeV.

The mass attenuation coefficient MAC was plotted against some selected energy values (0.060, 0.662, 1.173, and 1.333 MeV) while the MAC ranges from 0.0546 to 1.1890 cm^2^/g (see Fig. 7). At the lowest energy level of 0.060 MeV where the interaction is predominantly photoelectric, sample SR-CRT-50 shows the highest MAC value of 1.1890 cm^2^/g due to its high content of CRT components such as PbO, SiO_2_ etc., while sample SR-CRT-0 shows the least MAC value of 0.3029 cm^2^/g, other samples have MAC between these values. As the energy increased to 0.662 MeV, we observed a drastic decrease and convergence in the MAC values across all the samples due to Compton scattering interactions which is also affected by high atomic number, as shown in Fig. 7. Moreover, at the highest energy level (1.333 MeV) where its predominantly a region for pair production which is less affected by lower atomic number element, sample SR-CRT-0 shows the highest MAC value of 0.0551 cm^2^/g due to its zero content of CRT, indicating the highest attenuation strength as shown in the inset of Fig. 7, while sample SR-CRT-50 shows the least MAC (0.0546 cm^2^/g) indicating the least attenuation ability. While pair production is indeed proportional to Z, the observed trend in MAC values may be attributed to the influence of other factors such as structural composition, and the interaction of photons with the matrix components, which can modulate attenuation behavior in these samples.

**Fig. 8:**
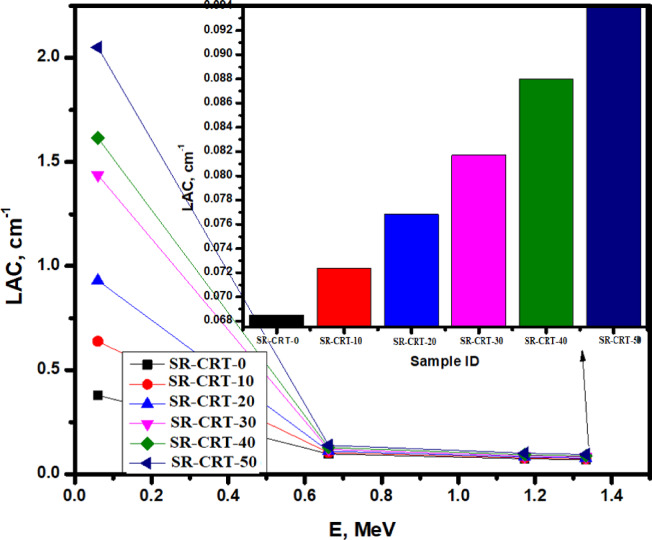
A chart of Linear Attenuation Coefficient LAC for all the studied samples as a function of Energy E in MeV.

Figure 8 shows the results of the linear attenuation coefficient for the prepared composites. At the lowest energy level of 0.060 MeV, we noticed that the LAC was very high for all the fabricated samples with sample SR-CRT-50 showing the highest LAC value of 2.051 cm^−1^ due to its highest composition of CRT components such as PbO, SrO and BaO, followed by sample SR-CRT-40 with a value of 1.616 cm^−1^, while sample SR-CRT-0 due to its least content of CRT has the lowest LAC with a value of 0.379 cm^−1^ (see the inset of Fig. 8). As the energy increased from 0.060 to 0.662 MeV, the LAC values dropped significantly for all the samples, for example, sample SR-CRT-50 decreased from 2.051 to 0.139 cm^−1^ showing the greatest decrease in LAC, while sample SR-CRT-0 decreased from 0.379 to 0.097 cm^−1^. Between 0.662 to 1.333 MeV, we noticed a small decrease in the LAC values for all the samples, and at the highest energy level of 1.333 MeV, sample SR-CRT-50 again shows the highest LAC with a value of 0.094 cm^−1^, while sample SR-CRT-0 shows the lowest LAC value. This trend shows that sample SR-CRT-50 has the highest attenuation ability at all energy levels.

**Fig. 9:**
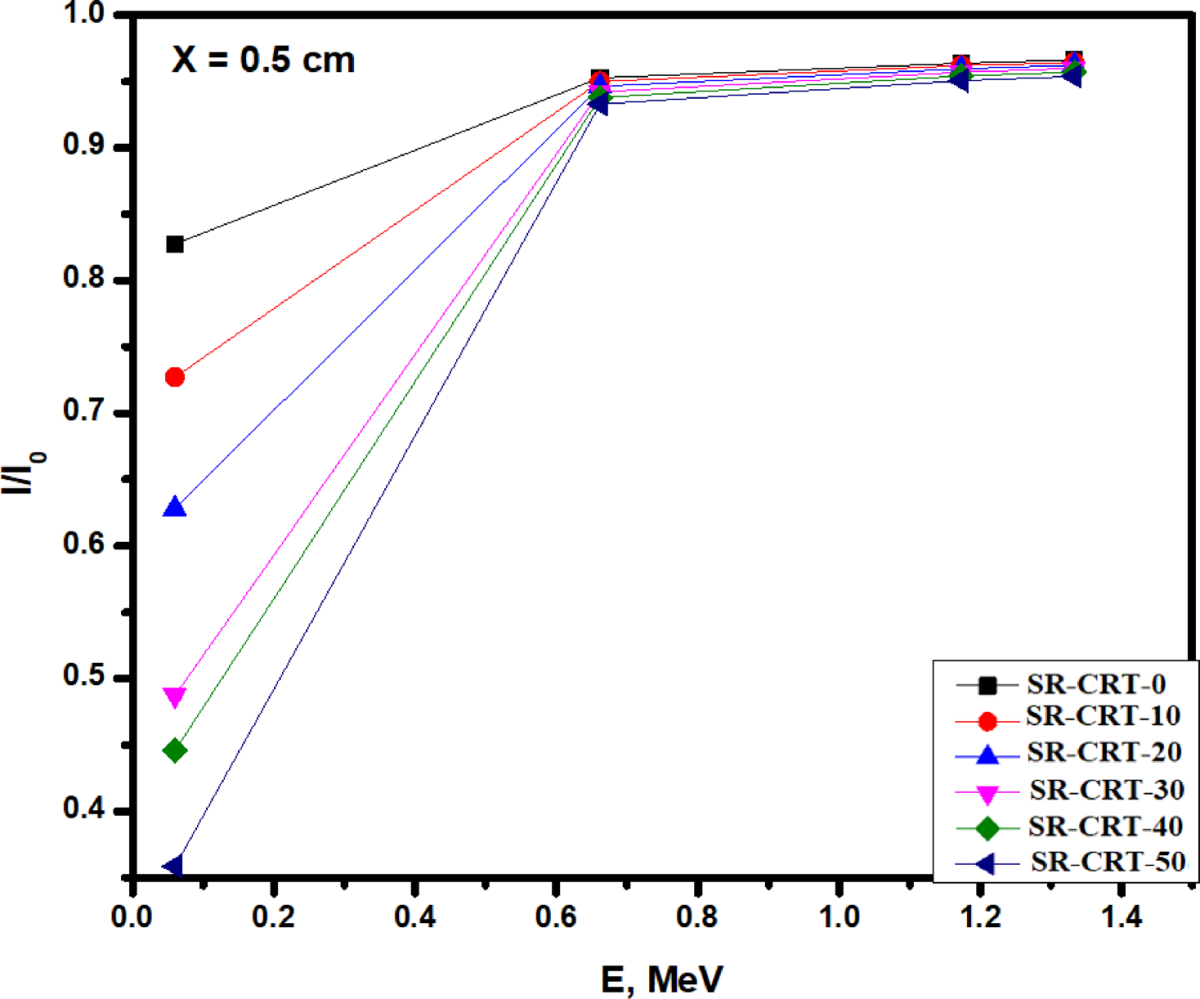
A chart of the Transmission Factor TF for all the studied samples as a function of Energy E in MeV at a thickness of 0.5 cm.

Figure 9 shows The TF values for the prepared composites at different sample thicknesses 0.5, 1, 1.5 and 2 cm. Clearly, the TF started with least values across all the samples at 0.060 MeV with sample SR-CRT-50 showing the least TF values due to its high CRT contents such as PbO, SrO and BaO, which greatly interact and block most the intensity of the incoming radiation, while sample SR-CRT-0 shows a higher TF values as a result of the least content of the CRT compositions. As the energy increased to 0.662 MeV, there is a significant increase in the TF value across all the samples due to the increase in the intensity of the incoming radiation. As an illustration, the TF value of sample SR-CRT-50 increased from 0.357 to 0.933, sample SR-CRT-0 increased from 0.828 to 0.953 while the other samples fall between the values of sample SR-CRT-0 and sample SR-CRT-50. At the highest energies (0.660–1.333 MeV), the TF appears to converge and the increment was small. As an illustration, sample SR-CRT-50 increased from 0.933 to 0.954, while sample SR-CRT-0 increased from 0.953 to 0.966, the other samples were in-between the SR-CRT-50 and SR-CRT-0 values. Consistently, sample SR-CRT-50 consistently shows lower TF values due to higher content of the composition of CRT such as PbO, SrO and BaO, making it the highest attenuating sample, while sample SR-CRT-0 shows the highest TF values, indicating the poorest shielding ability.

**Fig. 10:**
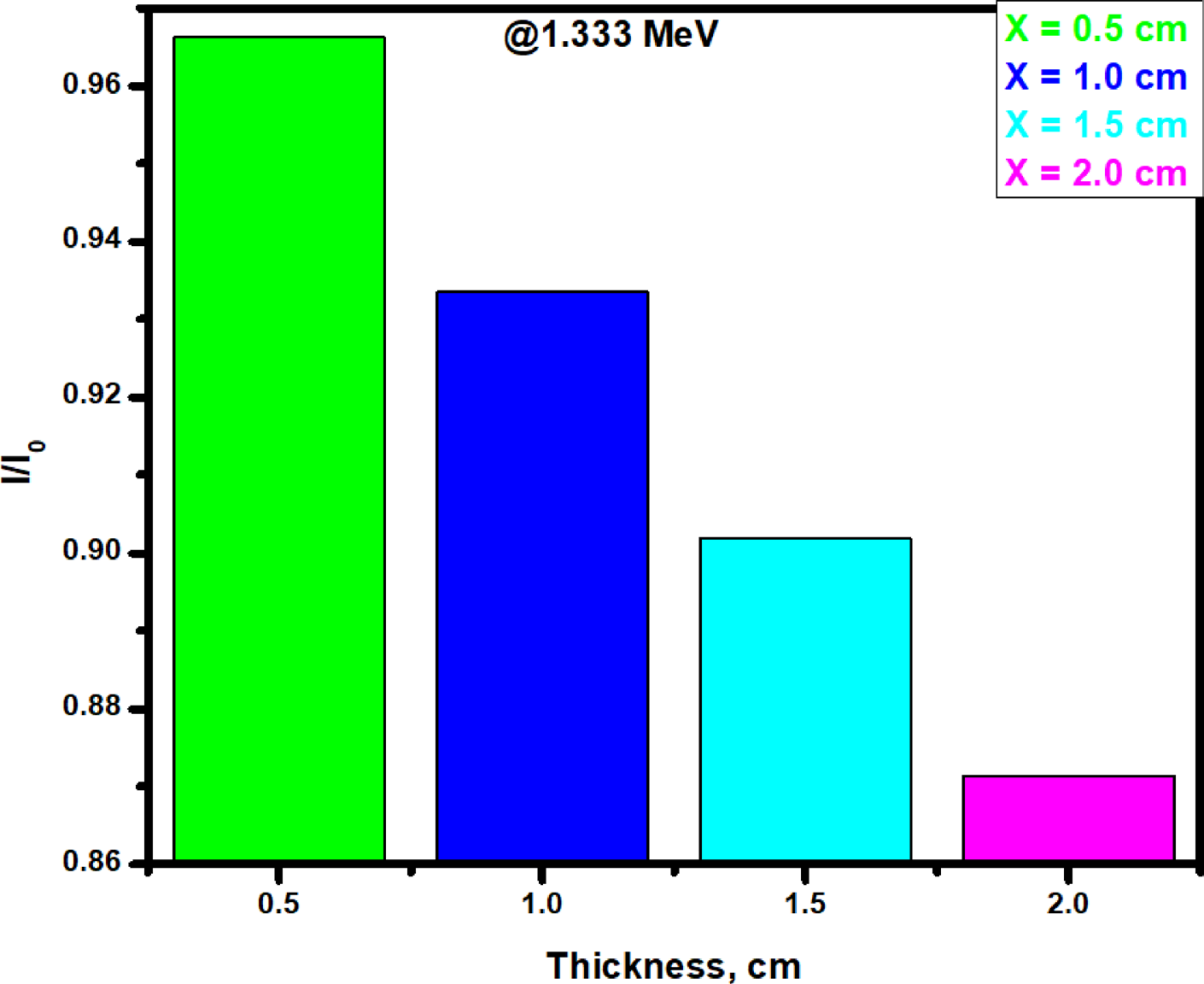
A chart of the Transmission Factor TF for sample CM-CRT-0 at thickness of 0.5, 1.0, 1.5, and 2.0 cm at an energy value of 1.333 MeV.

In Fig. 10, the TF for the SR-CRT-0 with different thicknesses was investigated at 1.333 MeV. At the highest energy level of 1.333 MeV and a thickness of 0.5 cm, SR-CRT-0 sample shows the highest TF value (0.966) due to its low CRT content (0%). Interestingly, as we increase the thickness of sample SR-CRT-0, we noticed a great decrease in the TF values which arises due to the increase in number of particles resulting in an increase in interaction between the composite molecules and the radiation. Due to the high number of particles as the thickness reaches 2.0 cm, the interaction between the composite particles and the radiation greatly increases causing the TF value to reduce drastically (0.871), signifying that radiation attenuation can be improve by increasing the thickness of the sample.

**Fig. 11:**
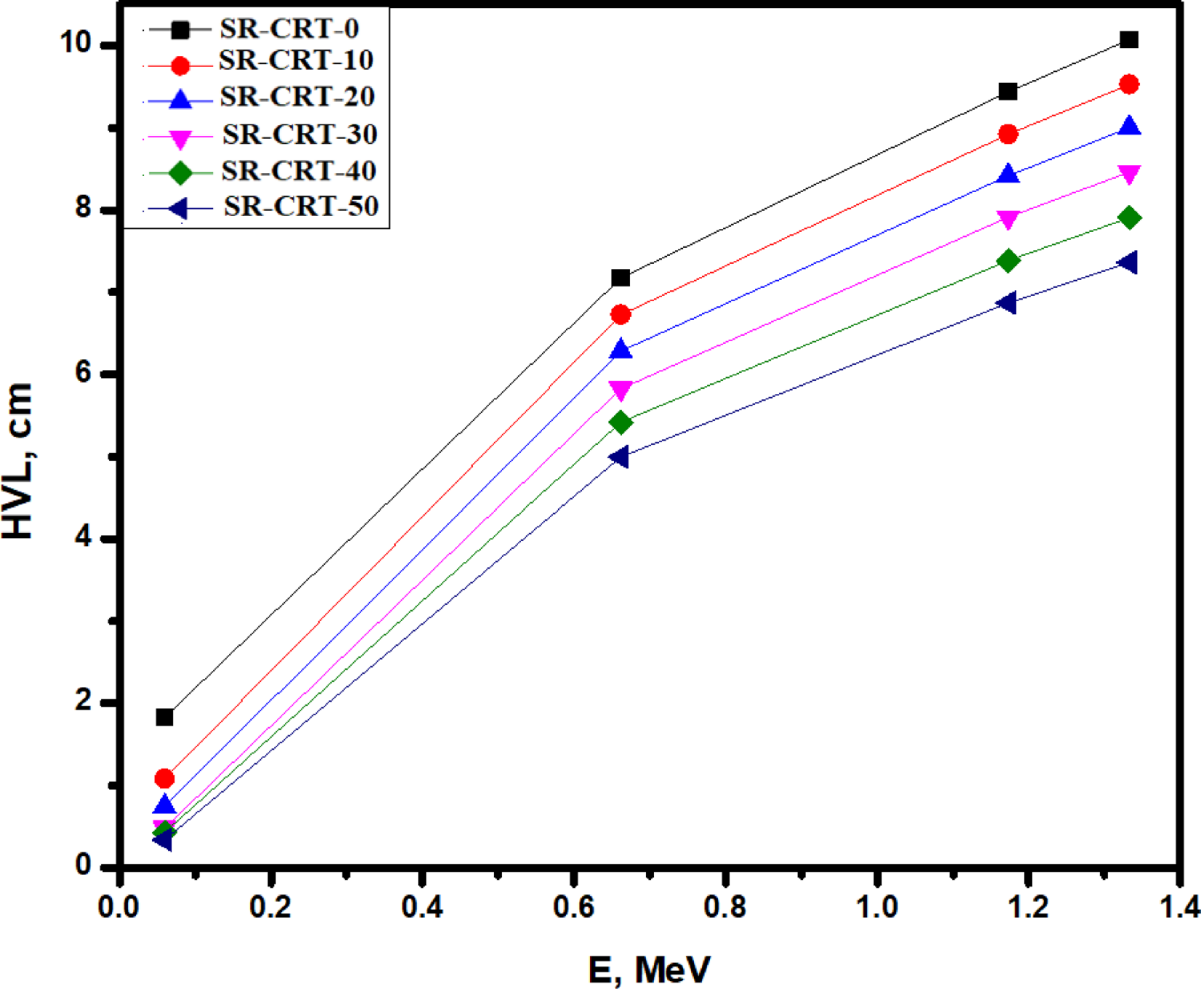
A chart of Half Value Layer HVL for all the studied composites as a factor of Energy E in MeV.

Figure 11 represents the HVL for the prepared composites. At the lower energy level of 0.060 MeV, three samples, sample SR-CRT-50, sample SR-CRT-40 and sample SR-CRT-30 shows a close HVL values of 0.338 cm, 0.429 cm, and 0.482 cm respectively due to the high content of CRT compositions in them, while the other samples show a clearly distinct HVLs. All the plots show highest slope due to the predominant photoelectric interaction between 0.060 to 0.662 MeV. As the energy increased from 0.662 to 1.173 MeV where Compton scattering interaction takes charge, the increase in the HVLs became less rapid with sample SR-CRT-50 showing the least increase in HVL (from 4.997 to 6.872 cm), while sample SR-CRT-0 shows the highest increase in HVL (from 7.176 to 9.437 cm). At the highest energy level of 1.333 MeV where pair production takes place, sample SR-CRT-50 again shows the least increase in HVL as shown in Fig. 11, while sample SR-CRT-0 shows the highest increase in HVL due to its lowest content of CRT compositions (0%). Therefore, sample SR-CRT-50 with its least HVLs throughout the energy spectrum indicated better attenuation ability than all the other samples especially sample SR-CRT-0 which happens to be the poorest radiation shielding material.

**Fig. 12:**
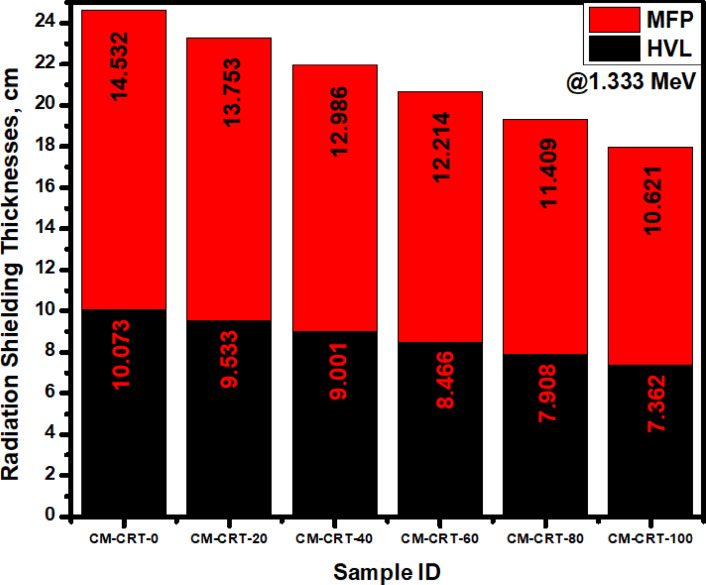
A comparison chart of HVL and MFP for all the fabricated samples at an energy value of 1.333 MeV.

From the plots in Fig. 12 we can see that sample SR-CRT-0 has the highest HVL (10.073 cm) and MFP (14.532 cm) values among all the studied samples. This is as a result of its less content of CRT compositions such PbO, SrO and BaO in its structure allowing for a deeper penetration of the radiation. This indicates the lowest shielding ability in sample SR-CRT-0. As we found in Fig. 10, Fig. 11 confirms that SR-CRT-50 has the lowest HVL and MFP due to the highest density of this composite.

**Fig. 13:**
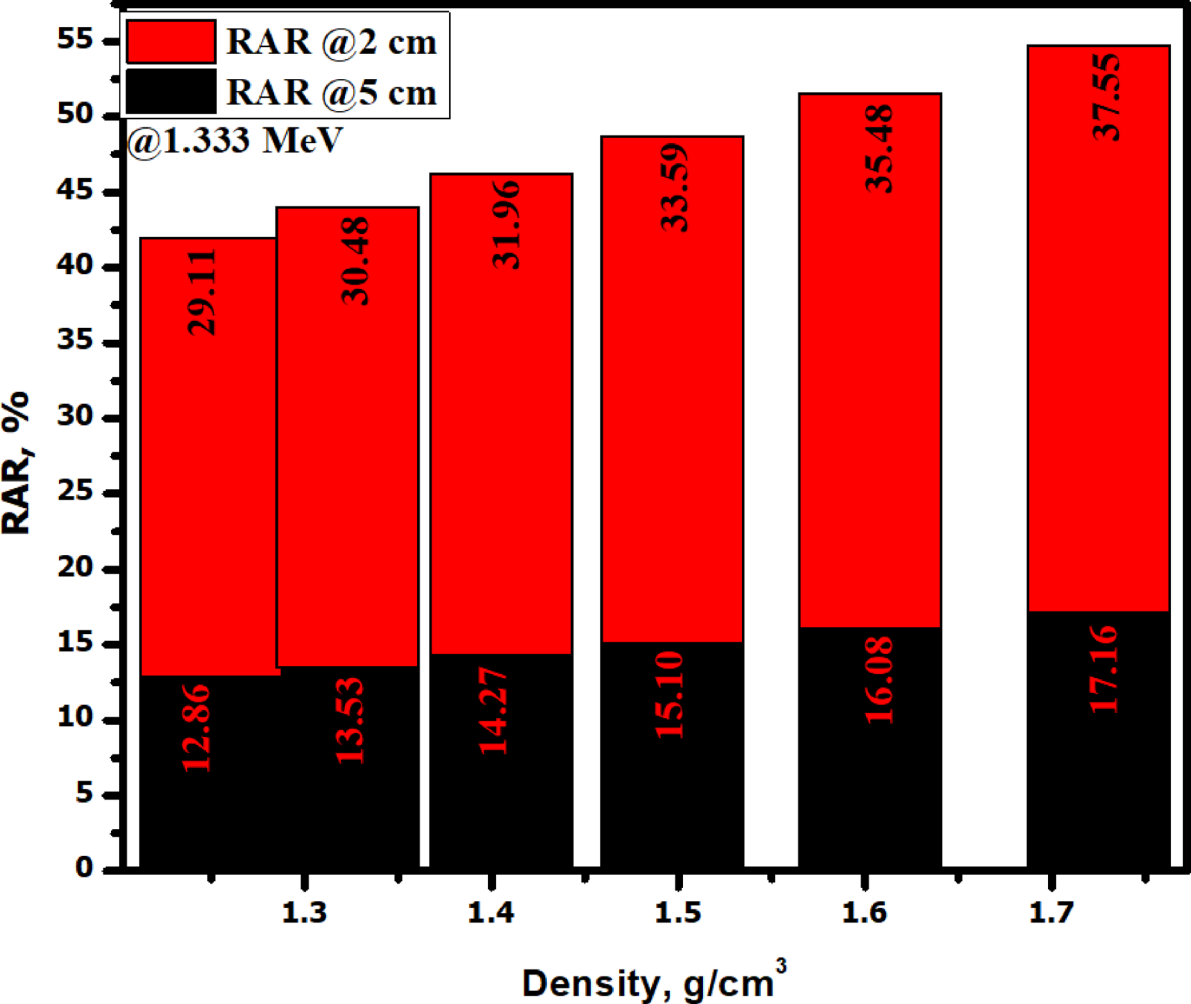
A comparison chart of the Radiation Attenuation Ratio at two different thicknesses (2 and 5 cm) for all the fabricated samples at an energy value of 1.333 MeVA.

Figure 13 compared the radiation attenuation ratio (RAR) at two different thicknesses (2 and 5 cm) for all the fabricated samples at an energy value of 1.333 MeV. Clearly, samples that are less dense (e.g. sample SR-CRT-0 and SR-CRT-10) shows the lowest RAR values at both thickness (29.11% at 2 cm and 12.86% at 5 cm for sample SR-CRT-0, and 30.48% at 2 cm and 13.53% at 5 cm), indicating the least protection efficiency in these samples. As the density increases, we noticed an increase in RAR values, as an illustration, sample SR-CRT-50 with highest density shows the highest RAR value as well (37.55% at 2 cm, and 17.16% at 5 cm) indicating the highest attenuation ability.

**Fig. 14:**
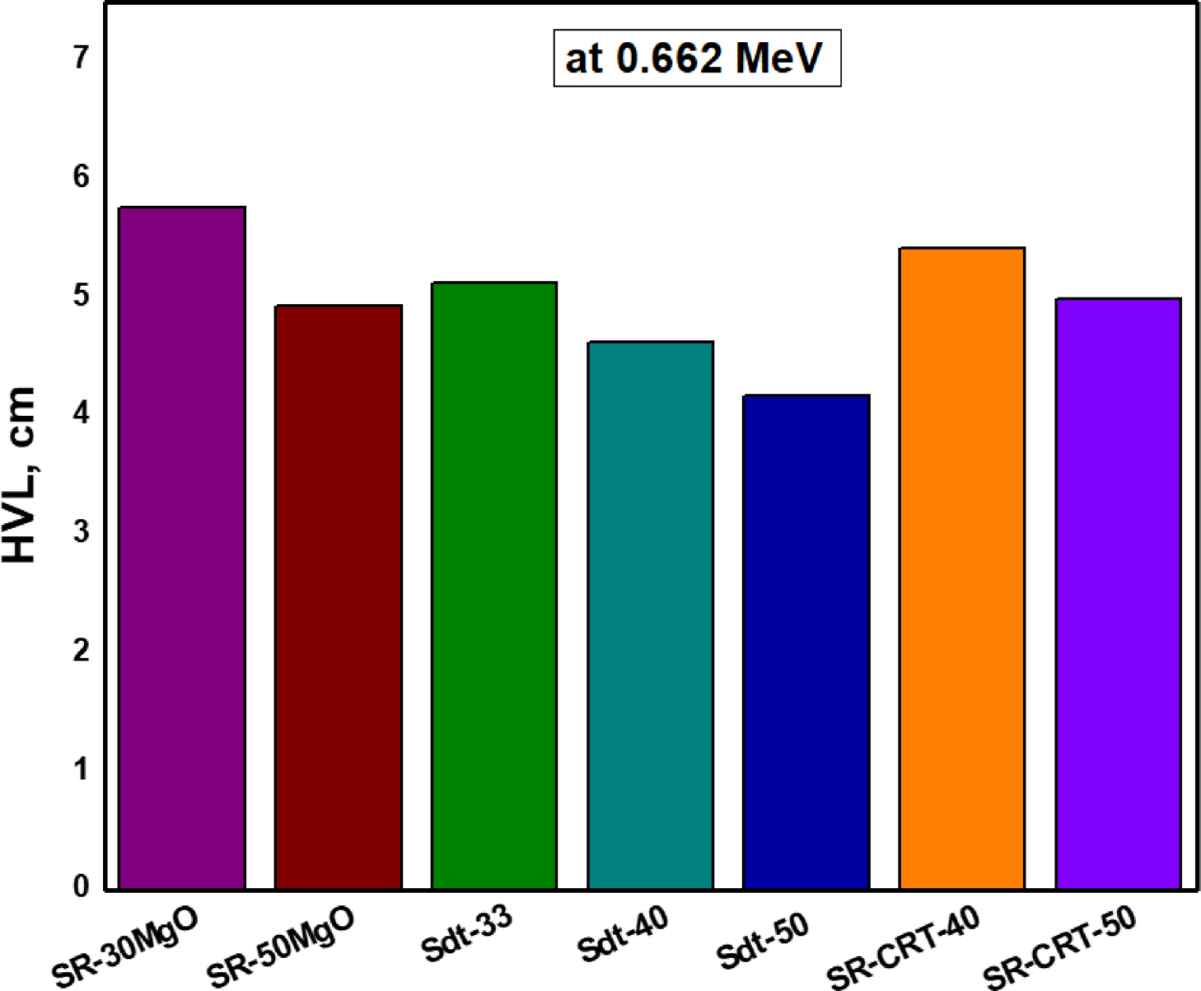
A comparison of present composites with related studies at 0.662 MeV.

Figure 14 compared the composites with the highest attenuation performance in the presrnt study (SR-CRT-40 and SR-CRT-50) with related published silicon composites such as SR-30MgO and SR-50MgO^42^ and Sdt-33, Sdt-40 and Sdt-50 or SR-Fe_2_O_3_ composites^43^. The results showed that the studied compounds have the ability to act as flexible shielding materials against photons, which is almost comparable to the composites doped with Fe_2_O_3_ and MgO.

## Conclusions

The present research investigated the radiation protection performance of a novel composite made of silicon rubber and CRT glass recycled from e-waste. Silicon rubber was utilized as a matrix and filled with varying amounts of CRT glass (0% to 50%). The radiation attenuation parameters of the composite samples (SR-CRT-0, SR-CRT-10, SR-CRT-20, SR-CRT-30, SR-CRT-40, and SR-CRT-50) were assessed experimentally by HPGe detector, and different sources of energy (1.173 & 1.333 MeV from Co-60, 0.66 MeV from Cs-137, and 0.06 MeV from Am-241). At 0.06 MeV, SR-CRT-50 showed the best radiation-interaction response with maximum LAC (2.051 cm^−1^), maximum MAC (1.1890 cm^2^/g), minimum HVL (0.338 cm) and minimum TF (0.357). This is attributed to the high content of CRT (50%) which contains PbO, SrO and BaO; these metal oxides can block the majority of the incoming radiation. On the contrary, at 1.333 MeV, the SR-CRT-0 sample showed the poorest radiation shielding performance with the highest TF value (0.966) as it doesn’t include any CRT (0%). The evaluation mechanical performance of the prepared SR-CRT samples proved that adding CRT glass didn’t diminish the mechanical features of the silicone rubber. The stress–strain data indicated that using lower concentrations of CRT glass can keep the balance between strength and flexibility. On the other hand, using higher concentrations of CRT can lead to higher mechanical strength but at the expense of flexibility. Considering thermal performance, our findings showed that adding CRT-glass to silicon rubber enhanced the thermal properties of the silicone rubber. In other words, the higher the CRT content in silicone rubber, the better the thermal properties. Conclusively, the proposed unique composite proved its promising potential of being used in various radiation shielding applications where flexibility and lightweight are required such as in medical imaging, aerospace and military settings. Future studies should examine incorporating CRT glass into other matrices, such as different types of polymers, alloys, ceramics, and building materials, in order to completely understand the properties of CRT glass as a filler material in radiation shielding composites.

## Data Availability

“The data that support the findings of this study are available from the corresponding author upon request”.
